# Identification of prognostic and therapeutic biomarkers in type 2 papillary renal cell carcinoma

**DOI:** 10.1186/s12957-022-02836-3

**Published:** 2023-03-16

**Authors:** Yue Wang, Xi Tian, Shu-Xuan Zhu, Wen-Hao Xu, Aihetaimujiang Anwaier, Jia-Qi Su, Hua-Lei Gan, Yuan-Yuan Qu, Jian-Yuan Zhao, Hai-Liang Zhang, Ding-Wei Ye

**Affiliations:** 1 Department of Urology, Fudan University Shanghai Cancer Center, State Key Laboratory of Genetic Engineering, Collaborative Innovation Center for Genetics and Development, School of Life Sciences, Fudan University, Shanghai, 200433 China; 2 Department of Oncology, Shanghai Medical College, Fudan University, Shanghai, 20032 People’s Republic of China; 3 Department of Endocrine and Breast Surgery, The First Affiliated Hospital of Chongqing Medical University, Chongqing Medical University, Chongqing, 400016 China; 4 Department of Pathology, Fudan University Shanghai Cancer Center, Shanghai, 200032 China

**Keywords:** Type 2 papillary renal cell carcinoma, Prognosis, mTOR inhibitor, Biomarker

## Abstract

**Background:**

Papillary renal cell carcinoma (PRCC) can be divided into type 1 (PRCC1) and type 2 (PRCC2) and PRCC2 share a more invasive phenotype and worse prognosis. This study aims to identify potential prognostic and therapeutic biomarkers in PRCC2.

**Methods:**

A cohort from The Cancer Genome Atlas and two datasets from Gene Expression Omnibus were examined. Common differentially expressed genes (DEGs) were screened and potential biomarkers were explored by using Kaplan–Meier method and cox regression analysis. Functional enrichment analysis was utilized to evaluate the potential biological functions. Tumor infiltrating immune cells were estimated by CIBERSORT algorithm. Ninety-two PRCC2 samples from Fudan University Shanghai Cancer Center were obtained, and immunostaining was performed to validate prognostic and therapeutic significance of the potential biomarker.

**Results:**

PRCC2 has worse overall survival and shares distinct molecular characteristics from PRCC1. There was significant higher expression level of Targeting protein for Xklp2 (TPX2) in PRCC2 compared with normal tissues. Higher expression level of TPX2 was significantly associated with worse overall survival in PRCC2 and kinesin family genes expression were found significantly elevated in high risk PRCC2. Abundance of tumor infiltrating M1 macrophage was significantly higher in PRCC2 and it was also associated with worse overall survival. In the FUSCC cohort, higher TPX2 expression was significantly correlated with worse overall and progression-free survival. Retrospective analysis indicated that mTOR inhibitor (everolimus) had greater efficacy in the high-risk group than in the low-risk group (overall response rate: 28.6% vs. 16.7%) and that everolimus had greater efficacy than sunitinib in the high-risk group (overall response rate: 28.6% vs. 20%).

**Conclusions:**

TPX2 was a prognostic and therapeutic biomarker in PRCC2. Higher abundance of tumor infiltrating M1 macrophage was significantly associated with worse overall survival in PRCC2. mTOR inhibitors may have good efficacy in patients with high-risk PRCC2.

**Supplementary Information:**

The online version contains supplementary material available at 10.1186/s12957-022-02836-3.

## Introduction

Renal cell carcinoma (RCC) is the third most common malignant tumor of the genitourinary system [1]. Clear cell RCC (ccRCC) represents approximately 70% of kidney cancer cases in adults [2]. Papillary renal cell carcinoma (PRCC) is the most common non–clear cell RCC (nccRCC), accounting for 10%–15% of RCCs [3]. Surgery is the first choice for RCC, and identifying the molecular mechanism of the tumor will provide a better overall assessment [4, 5]. Delahunt and Eble [6] characterized the histologic dissimilarities of PRCC and divided this malignancy into two subtypes (PRCC1 and PRCC2). Molecular analysis further clarified differences between the two subtypes. PRCC1 features gains in chromosomes 7, 17, 16 and 20 but loss of the Y chromosome [7]. MET pathway activation is frequently implicated in PRCC1 [8]. Conversely, PRCC2 has a more heterogenous spectrum of chromosomal gains and losses. It has been reported that 8q gains are especially related to the poor prognosis of PRCC2, and the NRF–ARE2 pathway was also revealed to be enriched in PRCC2 [9, 10]. Previous studies demonstrated that PRCC2 has significantly worse clinical outcomes than PRCC1 [11, 12]. In summary, PRCC2 differs from PRCC1 and features a more aggressive phenotype.

PRCC2 can be further divided into hereditary and sporadic types. The hereditary form is associated with biallelic inactivation of the gene encoding the Krebs cycle enzyme fumarate hydratase (FH), which leads to hereditary leiomyomatosis and RCC (HLRCC) syndrome, which is characterized by a high incidence of RCC, uterine leiomyoma, and cutaneous leiomyomatosis [13, 14]. Patients with HLRCC syndrome are also genetically susceptible to bladder cancer, collecting duct tumors, and adult Leydig cell tumors of the testes [15–17]. Sporadic PRCC2 (sPRCC2) accounts for most cases of PRCC2, and previous studies demonstrated that despite differences in genetic etiology, sPRCC2 shares many clinical and morphologic phenotypes with HLRCC syndrome [18]. The most prominent common biochemical feature of HLRCC syndrome and sPRCC2 is the continuous activation of NRF2, which is caused by intracellular fumaric acid accumulation attributable to fumarate hydratase (FH) inactivation [18], but the mechanism of NRF2 activation in sPRCC2 has not been determined.

Although rapid progress in medical science has facilitated the development of cancer therapy and multiple new drugs exert antitumor effects against ccRCC, problems remain in the management of PRCC2. Numerous clinical trials aimed to explore potential useful treatments for PRCC. Ravaud et al.[19] found that sunitinib was effective in the treatment of metastatic PRCC1 and PRCC2, but its efficacy was lower than that against metastatic ccRCC. In addition, Armstrong et al. [20] claimed that compared with everolimus, sunitinib improved PFS in patients with metastatic nccRCC. However, the results of these clinical trials including various targeted therapies and immunotherapies did not revolutionize the treatment of PRCC [21–24]. Because of its rarity and heterogeneity, there is little useful information regarding the rational clinical management of metastatic PRCC2.

Although OS is considered short in PRCC2, we also identified a subset of patients with histopathologically confirmed sPRCC2 and prolonged survival. It is important to clarify the mechanism responsible for the difference in survival. In this research, we focused on the molecular pattern of sPRCC2 and explored potential prognostic and therapeutic biomarkers in sPRCC2.

## Materials and methods

### Comparison of PRCC1 and PRCC2 in the cancer genome atlas (TCGA) cohort

Data for 77 patients with PRCC1 and 85 patients with PRCC2 and complete genetic alteration and clinical data were obtained from TCGA. Clinical information and genetic alterations in PRCC1 and PRCC2 were obtained from cBioPortal (https://www.cbioportal.org/). The Kaplan–Meier method was used to compare OS and disease-free survival (DFS) between the PRCC1 and PRCC2 groups. The chi-squared test and Kruskal–Wallis test were also applied to assess other clinical information including American Joint Committee on Cancer tumor stage, lymph node stage, metastasis stage, and serum calcium levels.

### Gene expression profiles of PRCC2 and differential gene expression analysis

Gene expression profiles and clinical information for patients with PRCC in TCGA were downloaded from https://portal.gdc.cancer.gov/. Germline mutation data in PRCC2 were obtained from the supplementary file of a previous study [25]. Because the molecular patterns and clinical behavior varied between patients with hereditary PRCC2 (patients with *FH* germline mutation) and sPRCC2, this study only focused on sPRCC2 (82 samples from TCGA, Table 1) and hereditary PRCC2 was excluded (two patients). The mutation patterns and corresponding gene expression patterns of these 82 sPRCC2 samples were obtained from cBioPortal. Two datasets containing expression profiles of sPRCC2 were downloaded from Gene Expression Omnibus: GSE26574 (https://www.ncbi.nlm.nih.gov/geo/query/acc.cgi?acc=GSE26574, contains 12 sPRCC2 samples) and GSE48352 (https://www.ncbi.nlm.nih.gov/geo/query/acc.cgi?acc=GSE48352, contains 19 sPRCC2 samples). The limma package [26] and GEO2R were used to explore differentially expressed genes (DEGs) between normal and sPRCC2 tissues from these three cohorts (adjusted ***p*** < 0.05 and fold change ≥ 2). A Venn diagram was applied to identify the overlapping upregulated and downregulated DEGs.

**Table 1:** Clinicopathological characteristics of 82 patients diagnosed with sporadic type-2 papillary renal cell carcinoma (without FH germline mutation) from TCGA cohort

Characteristics	Entire cohort (*N* = 82)
N (%)	
Age	
< 70 years	51 (62.2)
≥ 70 years	30 (36.6)
Censored	1 (1.2)
Gender	
Male	58 (70.7)
Female	24 (29.3)
Laterality	
Left	46 (56.1)
Right	36 (43.9)
pathologic T stage^a^	
T1 – T2	58 (70.7)
T3 – T4	24 (29.3)
pathologic N stage^a^	
N0	15 (18.3)
N1	11 (13.4)
N2	3 (3.7)
NX & Censored	53 (64.6)
pathologic M stage^a^	
M0	31 (37.8)
M1	2 (2.4)
MX & Censored	49 (59.8)

^a^TNM scoring system: Tumor size, Lymph Nodes affected, Metastases

### Identifying potential prognostic biomarkers in sPRCC2

Protein–protein interaction (PPI) networks of the overlapping upregulated and downregulated DEGs were separately constructed using the Search Tool for the Retrieval of Interacting Genes (http://string-db.org, version 10.0) online database. MCODE (version 1.4.2) [27], a Cytoscape plug-in [28], was used to identify the most significant hub genes in the PPI network. Univariate regression analysis was utilized to assess the prognostic value of potential biomarkers. Clinicopathological parameters were also taken into analysis. Biomarker with minimum *p* value in univariate regression was selected for further analysis.

### Potential biological function changes in high sPRCC2

Univariate regression analysis in TCGA cohort indicated that some DEGs may serve as prognostic biomarker. Multivariate regression analysis was not used due to the large number of deletions of clinical data in TCGA cohort. C-index was used to evaluate the performance of the potential biomarkers and the results were listed in Table 2. C-indexes indicated that TOP2A may be the most possible biomarker, but a previous study has explored the potential significance of TOP2A in PRCC [29]. Thus, we focused on TPX2 which is also of high C-index in prognostic model. As TPX2 is of prognostic significance, we divided sPRCC2 into low and high risk group based on expression level of TPX2 (cutoff was set as median expression). Differentially expressed genes between high and low risk sPRCC2 were explored and PPI network was constructed. Functional enrichment analysis based on gene ontology (GO) [30] and Kyoto Encyclopedia of Genes and Genomes (KEGG) [31] data bases was utilized to explore the potential biological functions of the DEGs by using ClusterProfiler package [32]. Expression levels of kinesin family genes were compared between low and high risk sPRCC2 and Kaplan–Meier method was applied to assess the prognostic value (cutoff was set according to survminer package). Gene set enrichment analysis were also utilized to explore potential biological changes.

**Table 2:** Univariate regression analysis and C-index of potential biomarkers in sPRCC2

Gene	Hazard ratio	*p*-value	C-index	Standard error
TPX2	1.287621	1.25E-06	0.838802	0.038811
TOP2A	1.197488	1.96E-06	0.850214	0.033834
KIF4A	2.246519	1.66E-05	0.804565	0.046405
RRM2	1.313137	2.71E-05	0.796006	0.049031
CCNB2	1.454794	4.01E-05	0.830243	0.045104
UBE2C	1.091226	5.80E-05	0.817404	0.048132
TTK	2.575851	6.21E-05	0.770328	0.056485
AURKA	1.593763	7.89E-05	0.718973	0.083864
BUB1B	2.243341	0.000102	0.756063	0.054928
PTTG1	1.17317	0.00019	0.78174	0.055651
CCNB1	1.162073	0.000209	0.696148	0.077868
MELK	2.008113	0.000514	0.708987	0.063193
CDC20	1.189536	0.000619	0.767475	0.051612
SUCNR1	1.015183	0.000681	0.741797	0.083578
NUSAP1	1.126137	0.003866	0.760342	0.051079
PTGER3	1.939939	0.007874	0.690442	0.07373
S1PR3	1.378566	0.041071	0.67903	0.078769

### Tumor microenvironment evaluation

As tumor microenvironment (TME) plays a key role in tumorigenesis and development and may be associated with patients’ prognosis, we explored the TME of PRCC2 by using bioinformatic tools. CIBERSORT [33] is a deconvolution algorithm that uses a set of gene expression values (corresponding to a "signature matrix" of 547 genes) to accurately estimate the composition of immune cells in tumor sample data. To explore the proportion of 22 kinds of tumor infiltrating immune cells (TIICs) in PRCC2 samples, the expression profile was normalized, and then R software was used to run the CIBERSORT algorithm with the number of permutations was set to 1000. The bar chart was drawn to show the composition of TIICs of each sample, correlations between TIICs abundance and biomarker expression was discussed and heat map was drawn. To further explore the biomarker’s potential impact on TME, we estimated the correlation between various immunomodulatory gene and biomarker.

### Validating the potential biomarkers in the FUSCC cohort

This study included 92 patients (clinical information is listed in Table 3) with histopathologically confirmed sPRCC2 (positive staining for FH) who underwent surgical treatment at FUSCC between 2009 and 2019, and tumor specimens were obtained with informed consent. Immunostaining of TPX2, KIF20A was performed using rabbit monoclonal anti-TPX2 antibody (Cat.ab270612, Abcam, USA) and Rabbit polyclonal to KIF20A (cat.15911–1-AP, Proteintech, USA). Positive or negative staining for a certain protein on a formalin-fixed, paraffin-embedded slide was independently assessed by two experienced pathologists. The staining intensity level was graded as follows: 0, no staining; 1, weak staining; 2, moderate staining; and 3, strong staining. The extent of staining ranged 0–4 based on the percentage of immunoreactive tumor cells (0%, 1%–25%, 26%–50%, 51%–75%, 76%–100%). The overall immunohistochemistry (IHC) score was obtained by multiplying the staining intensity by the extent of staining. IHC scores of 0–3 represented low risk, and scores of 4–12 indicated high risk. Then, the Kaplan–Meier method was used to compare OS and PFS between the groups.

**Table 3:** Clinicopathological characteristics of 92 patients diagnosed with sporadic type-2 papillary renal cell carcinoma (FH-IHC: positive) from FUSCC cohort

Characteristics	Entire cohort (*N *= 92)
N (%)	
Age	
< 70 years	81(88.0)
≥ 70 years	11 (12.0)
Gender	
Male	61 (66.3)
Female	31 (33.7)
Laterality	
Left	52 (56.5)
Right	40 (43.5)
Tumor size	
< 4 cm	34 (37.0)
≥ 4 cm	58 (63.0)
pathologic T stage^a^	
T1 – T2	68 (73.9)
T3 – T4	24 (26.1)
pathologic N stage^a^	
N0	57 (62.0)
N1	25 (27.2)
NX	10 (10.9)
pathologic M stage^a^	
M0	50 (54.3)
M1	24 (26.1)
MX	18 (19.6)
Fuhrman nuclear grade	
I-II	27 (29.3)
III-IV	65 (70.7)

^a^TNM scoring system: Tumor size, Lymph Nodes affected, Metastases

### Validation of the therapeutic significance of the biomarker

In total, 24 patients in the FUSCC cohort had a pathologically confirmed diagnosis of metastatic sPRCC2. We retrospectively collected the baseline characteristics, treatment details, and clinical outcomes of these patients by reviewing their electronic medical records, and the details were verified by two investigators. In the low-risk group, six patients received everolimus as first-line therapy. In the high-risk group, seven patients were treated with everolimus as the first-line therapy, and five patients were treated with sunitinib as the first-line therapy. Radiologic assessment was performed according to RECIST 1.1 criteria[34] to classify the best response to treatment as complete response (CR), partial response (PR), stable disease (SD), or progressive disease (PD).

## Results

### PRCC2 was more aggressive than PRCC1

**Fig. 1:**
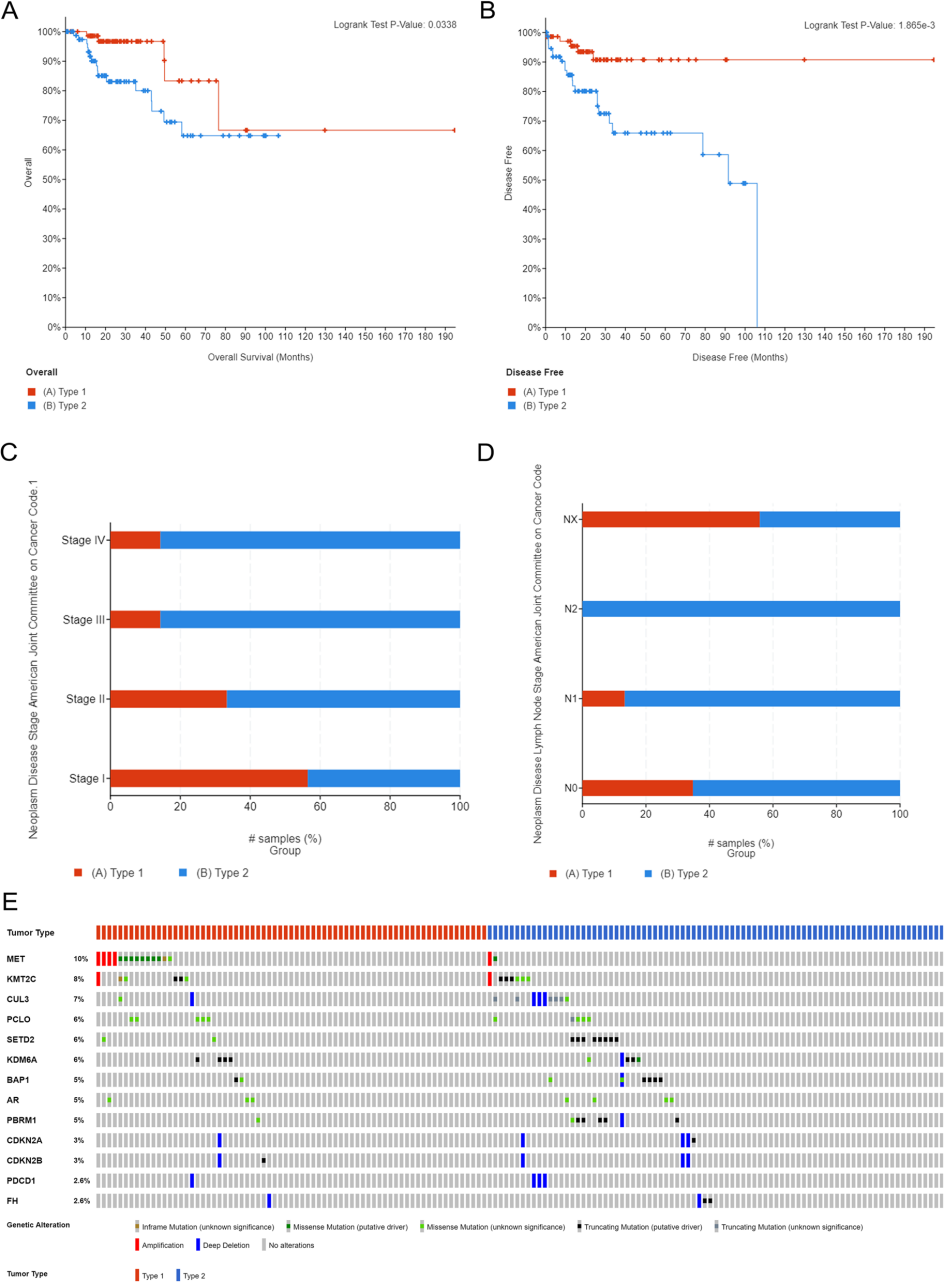
Overall survival and disease-free survival of PRCC2 compared with PRCC1 (**A**-**B**). Tumor stage and lymph node stage of two kinds of PRCC (**C-D**). Mutation landscape of PRCC (**E**)

Both OS and DFS were shorter in patients with PRCC2 than in those with PRCC1 (both *p* < 0.05, Fig. 1A–B), and the chi-squared test indicated that PRCC2 was often correlated with a higher tumor stage and lymph node stage (Fig. 1C–D). The somatic mutation pattern between PRCC1 and PRCC2 was diverse (Fig. 1E). PRCC1 had higher frequencies of *KMT2C* and *PCLO* mutation, whereas the most characteristic somatic alteration in PRCC1 was *MET* mutation. However, *MET* mutation was only detected in two PRCC2 samples. Meanwhile, PRCC2 had higher frequencies of *CUL3*, *SETD2*, and *PBRM1* mutation. In summary, PRCC2 is more aggressive and shares distinct molecular characteristics from PRCC1.

### Some common genes may play a key role in the malignant phenotype of sPRCC2

**Fig. 2:**
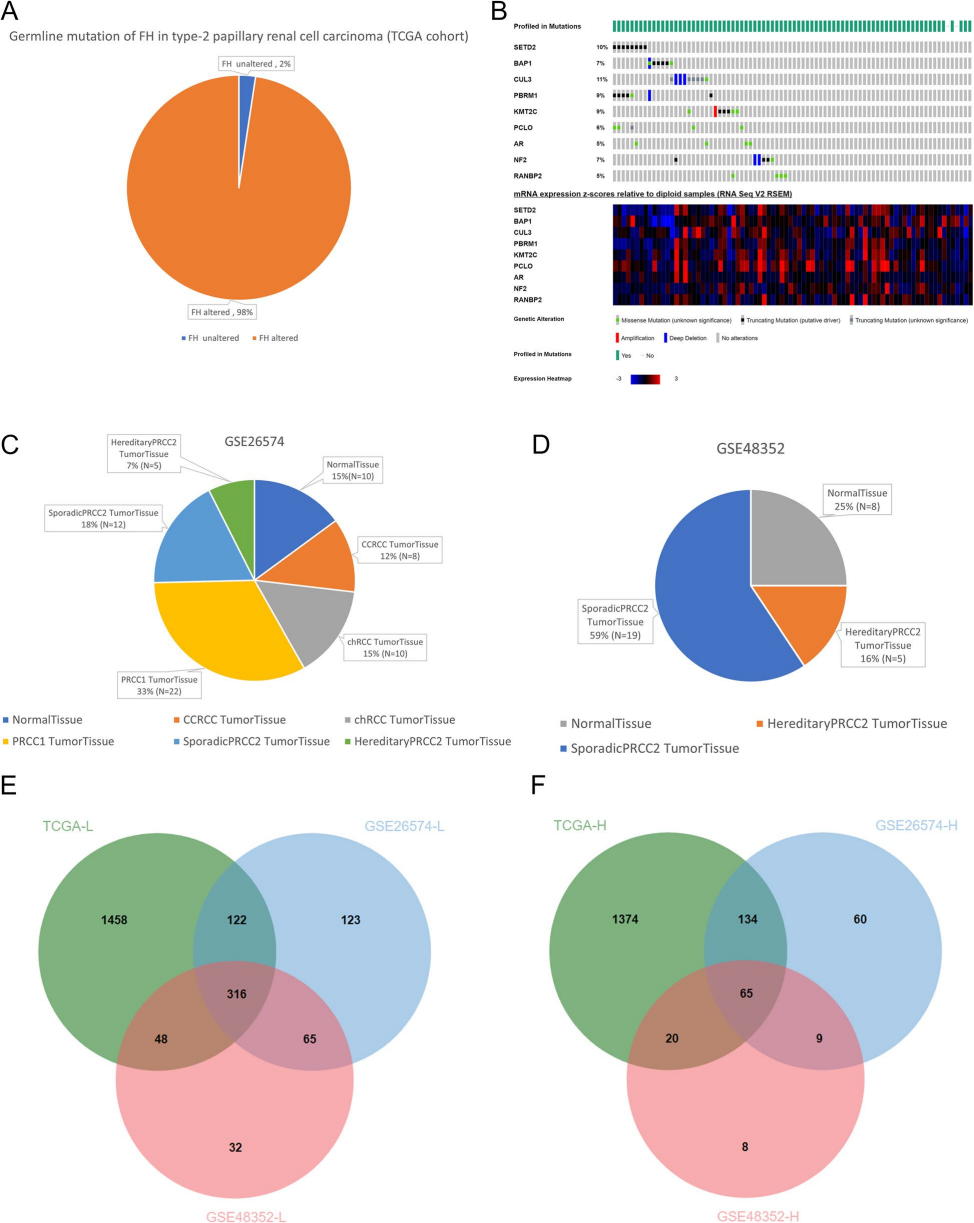
Germline FH mutation frequency of PRCC2 in TCGA cohort (**A**). Common pathologic variants and corresponding mRNA expression in sPRCC2 (**B**). Data composition of two data sets including GES26574 and GSE48352 (**C-D**). Veen diagrams of common down-regulated genes and common up-regulated genes in sPRCC2 (**E–F**)

TCGA cohort included samples from two patients with PRCC2 as indicated by *FH* germline mutation, and the remaining 82 patients with PRCC2 were grouped into the sPRCC2 cohort (Fig. 2A). *CUL3* mutation was most common in patients with sPRCC2, and *SETD2*, *PBRM1*, and *KMT2C* mutations were also common. These somatic mutations inevitably exerted influences on the gene expression pattern of sPRCC2 (Fig. 2B). The GSE26574 and GSE48352 datasets were also used to explore DEGs between sPRCC2 and normal tissues (Fig. 2C–D). In total, 316 downregulated genes and 65 upregulated genes were identified (Fig. 2E–F).

### Identify potential biomarkers in sPRCC2 and TPX2 was selected for further analysis

**Fig. 3:**
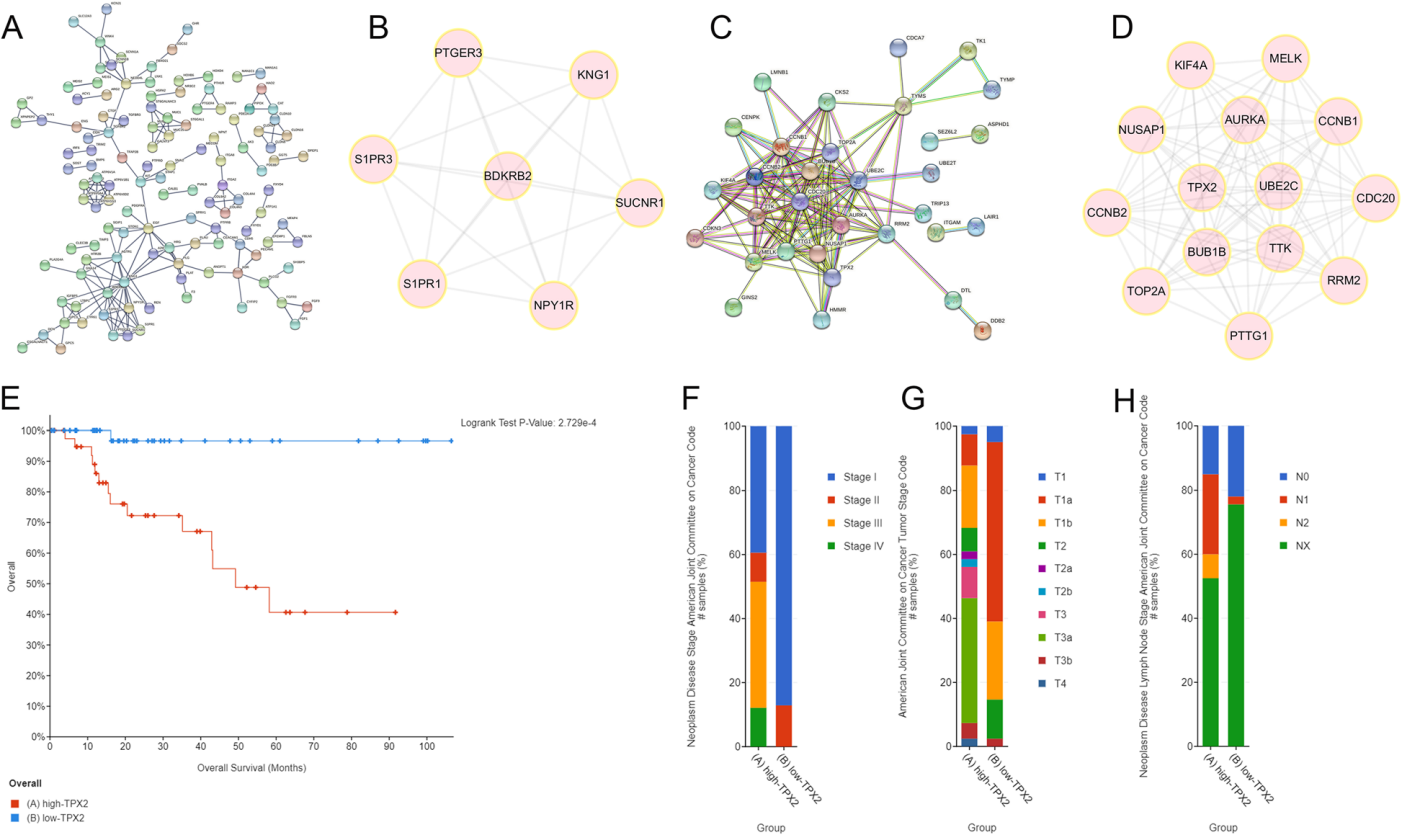
PPI network and hub genes of common down-regulated genes (**A-B**). PPI network and hub genes of common up-regulated genes (**C-D**). Overall survival curve of low and high TPX2 expression group in sPRCC2 (**E**). Clinical stage, T stage and lymph node stage of two groups (**F–H**)

PPI networks of downregulated genes and upregulated genes were constructed respectively (Fig. 3A, C). By using MCODE plugin in Cytoscape, downregulated hub genes (*BDKRB2*, *NPY1R*, *SUCNR1*, *KNG1*, *PTGER3*, *S1PR3*, *S1PR1*) and upregulated hub genes (*AURKA*, *TPX2*, *UBE2C*, *KIF20A*,*BUB1B*, *RRM2*, *CDC20*, *PTTG1*, *MELK*, *NUSAP1*, *TTK*, *CCNB2*, *CCNB1*, *TOP2A*) were screened (Fig. 3B, D). Univariate regression was used to assess the prognostic significance and the results were listed in Table 2. As TPX2 is the biomarker with minimum *p* value, further studies were focused on TPX2 and patients were divided into low and high risk group based on median expression level of TPX2. Survival curve indicated that higher expression level of TPX2 was significantly associated with sPRCC2 patients’ overall survival (Fig. 3E) and it is also correlated with higher clinical stage, tumor T stage and N stage (Fig. 3F-H). In summary, TPX2 could serve as a prognostic biomarker for sPRCC2.

### Differential gene expression analysis between low and high risk sPRCC2

**Fig. 4:**
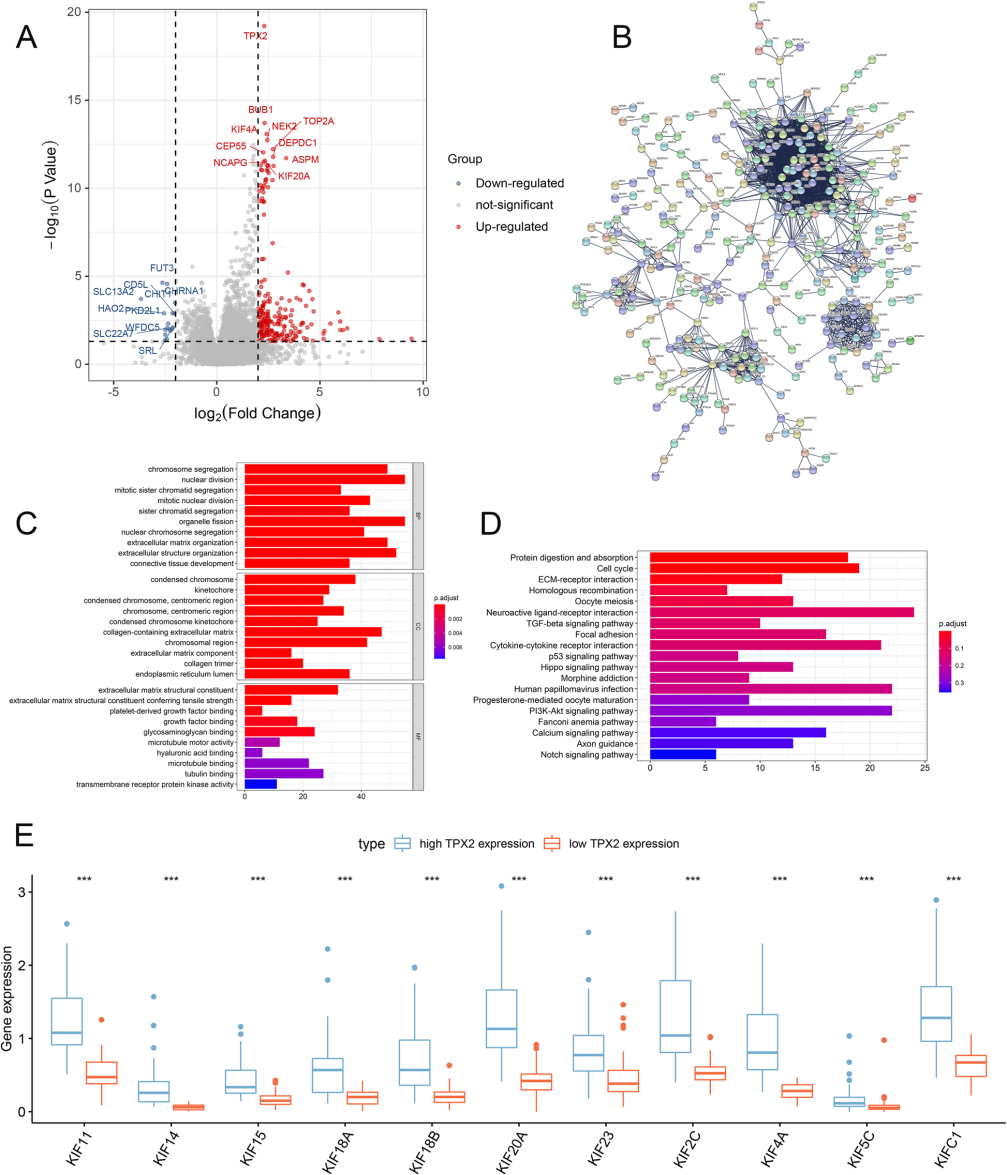
Volcano map of DEGs between high and low TPX2 expression groups (**A**). PPI network of the DEGs (**B**). GO and KEGG functional enrichment analysis of DEGs (**C-D**). Expression level of KIFs in low and high risk groups (**E**)

**Fig. 5:**
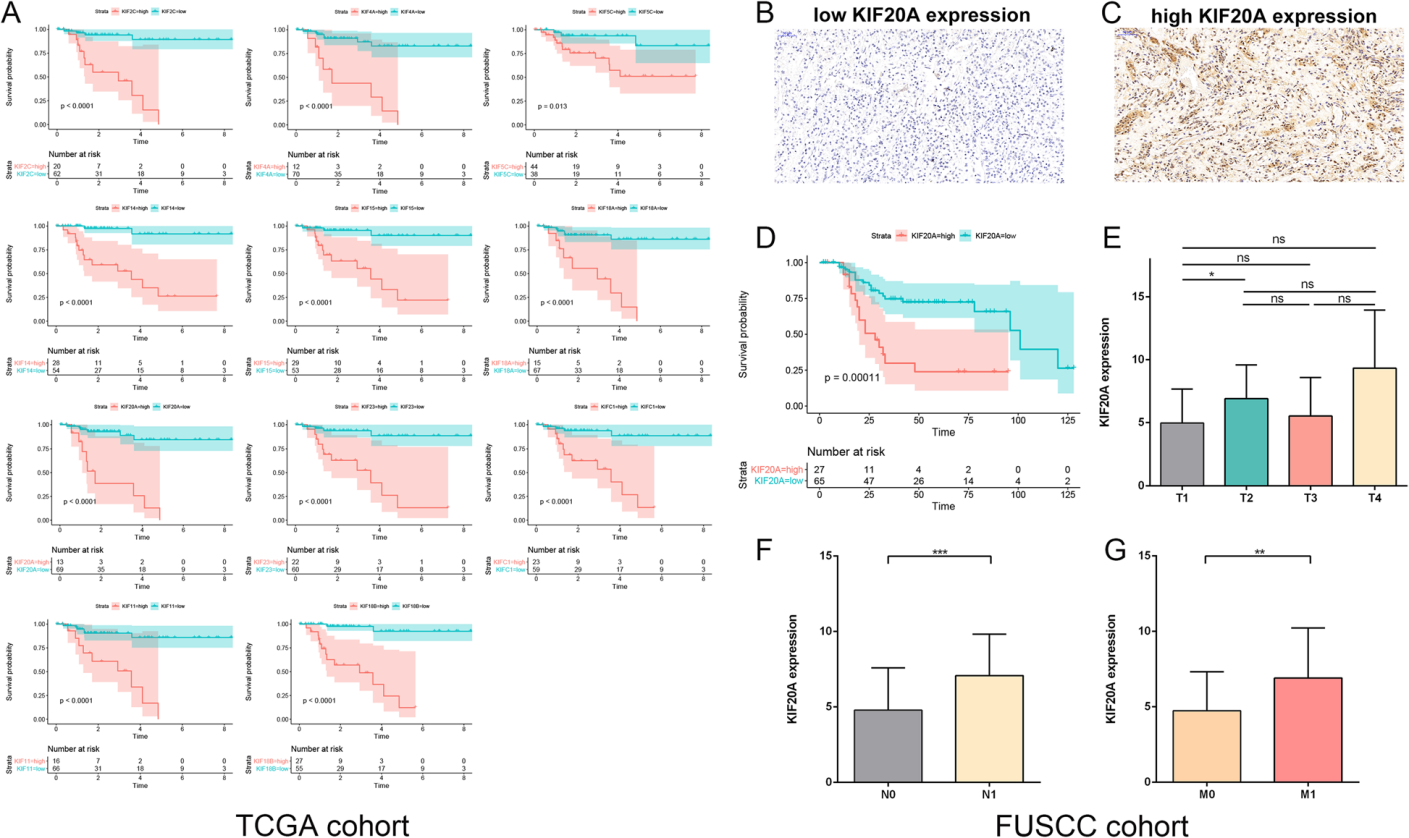
Survival curves of KIFs in sPRCC2 in TCGA cohort (**A**). Representative images of low and high KIF20A expression in FUSCC cohort (**B-C**). Survival curve and T, N, M stage of low and high KIF20A expression groups in FUSCC cohort (**D-G**)

By conducting differential gene expression analysis, 99 downregulated genes and 605 upregulated genes in high risk group were identified (Fig. 4A). PPI network of the DEGs were constructed (Fig. 4B) and functional enrichment analysis (Fig. 4C-D) indicated that the DEGs were mostly enriched in chromosome segregation, condensed chromosome, extracellular matrix structural constituent, protein digestion and absorption, etc. The full results of functional enrichment analysis were listed in supplementary materials. In addition, we found that 11 kinesin family genes were upregulated significantly in high risk sPRCC2 (Fig. 4E) and We hypothesize that the kinesin family may play key role in high risk PRCC. Kalan-Meier method indicated that these 11 kinesin family genes were all significantly associated with worse overall survival in sPRCC2 (Fig. 5A). As *KIF20A* is of minimum *p*-value and c-index in univariate regression analysis (Table 4), thus we further explored its potential prognostic significance in FUSCC cohort. IHC was used to detect expression level of KIF20A in FUSCC cohort and representative images of low and high expression were depicted (Fig. 5B, C). Survival analysis indicated that higher expression of KIF20A was significantly associated with worse overall survival, higher N stage and M stage in FUSCC cohort (Fig. 5D-G).

**Table 4:** Univariate regression analysis and C-index of kinesin family genes in sPRCC2

Gene	Hazard ratio	*p*-value	C-index	se
KIF20A	1.632578	3.84E-06	0.844508	0.039236
KIF18B	2.6608	2.88E-05	0.843081	0.036933
KIF4A	2.246519	1.66E-05	0.804565	0.046405
KIF2C	1.706585	8.25E-05	0.803138	0.042282
KIF14	14.78293	2.25E-05	0.794579	0.048578
KIFC1	1.712636	7.62E-06	0.78174	0.057966
KIF11	2.301793	2.57E-05	0.780314	0.050767
KIF15	18.09346	0.000126	0.768902	0.055698
KIF18A	3.529565	0.000276	0.768902	0.050087
KIF23	2.005171	0.000439	0.733238	0.062535
KIF5C	51.9065	0.020302	0.660485	0.081688

### M1 macrophage was significantly associated with worse overall survival in sPRCC2

**Fig. 6:**
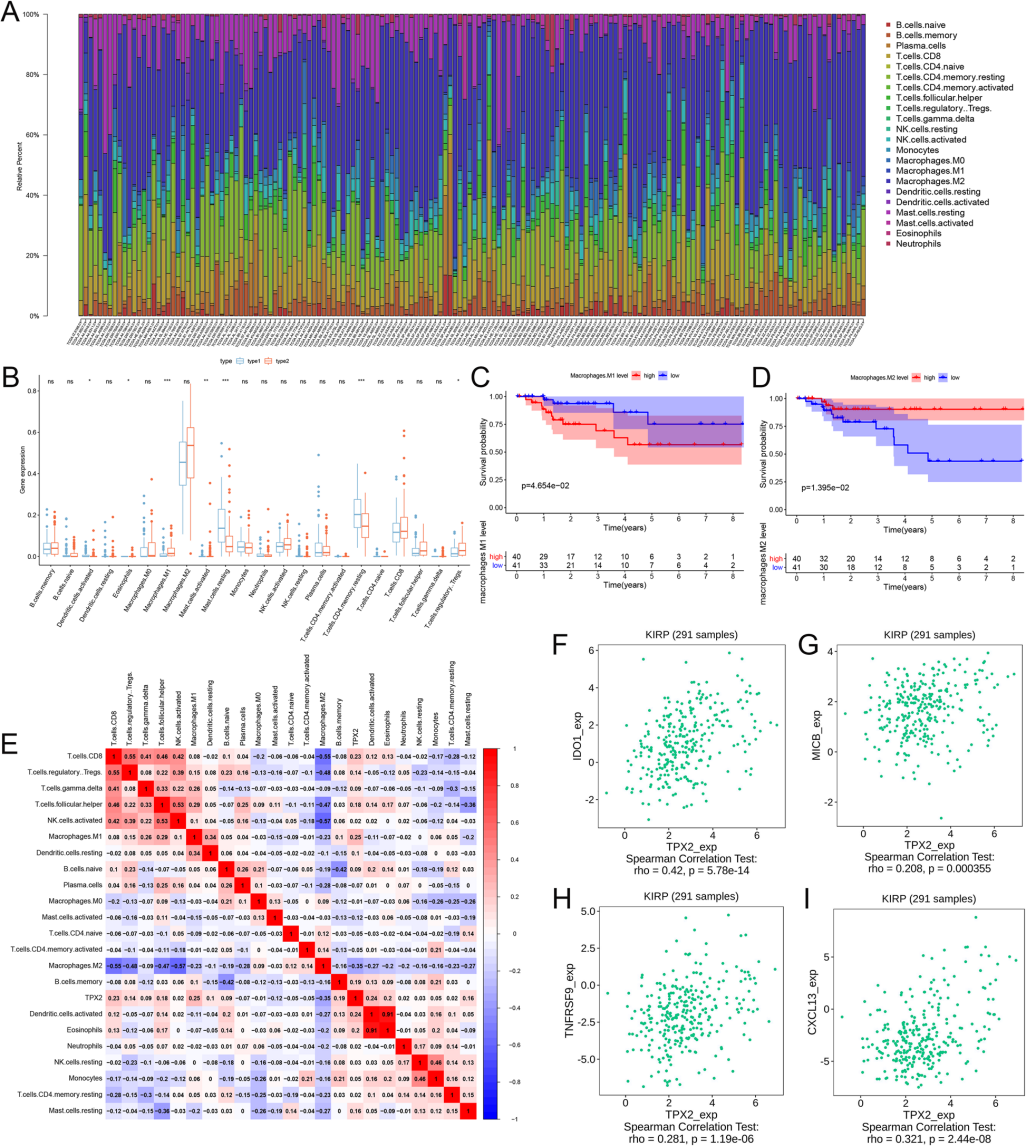
Bar plot of TIICs in PRCC and comparison of TIICs in PRCC1 and PRCC2 (**A-B**). Survival curves of M1 and M2 macrophage in sPRCC2 (**C-D**). Correlation heat map of various TIICs (**E**). Correlation analysis of TPX2 and various immune regulatory genes including IDO1, MICB, TNFRSF9, CXCL13 (**F-I**)

Abundance of tumor infiltrating immune cells (TIICs) in PRCC was evaluated by CIBERSORT algorithm and various TIICs increased in PRCC2 including M1 macrophage (*p* < 0.001), activated mast cells (*p* < 0.01), regulatory T cells (*p* < 0.05). While resting mast cells (*p* < 0.001) and resting memory CD4^+^ T cells (*p* < 0.001) were significantly lower in PRCC2 (Fig. 6A-B). Survival analysis (Fig. 6C-D) indicated that elevated infiltration of M1 macrophage was significantly associated with worse overall survival in PRCC2 (*p* < 0.05), while M2 macrophage was significantly associated with better overall survival (*p* < 0.05). Next, we aimed at exploring the association between TPX2 expression and TIICs. Correlation analysis (Fig. 6E) indicated that TPX2 was significantly associated with M1 macrophage abundance (correlation coefficient = 0.25) and activated dendritic cells (correlation coefficient = 0.24). Thus, TPX2 may play a subtle effect in TME and further exploration indicated (Fig. 6F-I) that TPX2 was significantly associated with various immune regulatory genes including IDO1, MICB, TNFRSF9 and CCL13 (rho = 0.42, 0.208, 0.281, 0.321). The results indicated that TPX2 may be associated with suppressive TME and thus weaken anti-tumor immunity.

### TPX2 could serve as prognostic and therapeutic biomarker in FUSCC cohort

**Fig. 7:**
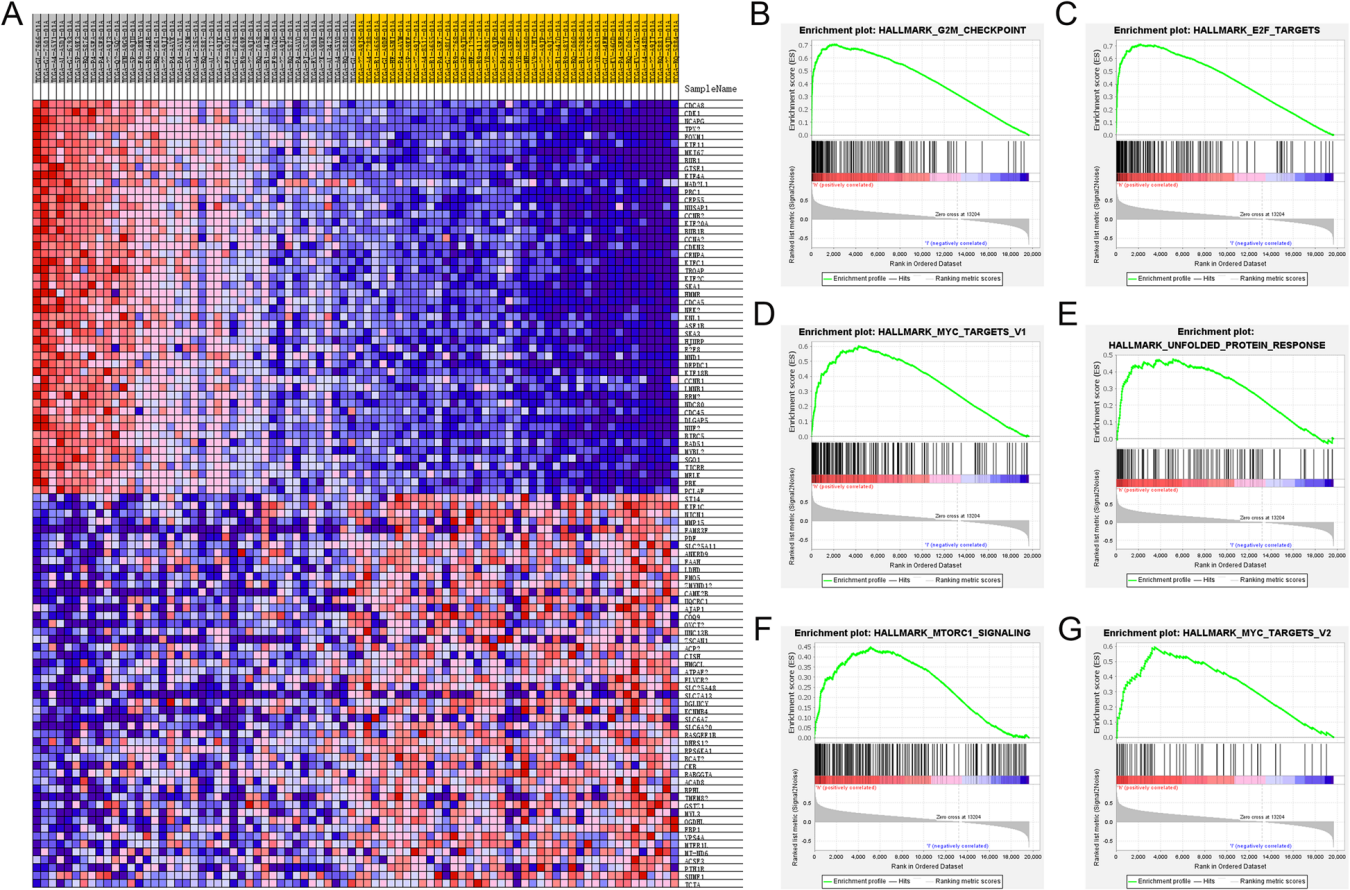
Global heat map of GSEA between high and low risk group in sPRCC2. Various significant biological processes in high risk sPRCC2

**Fig. 8:**
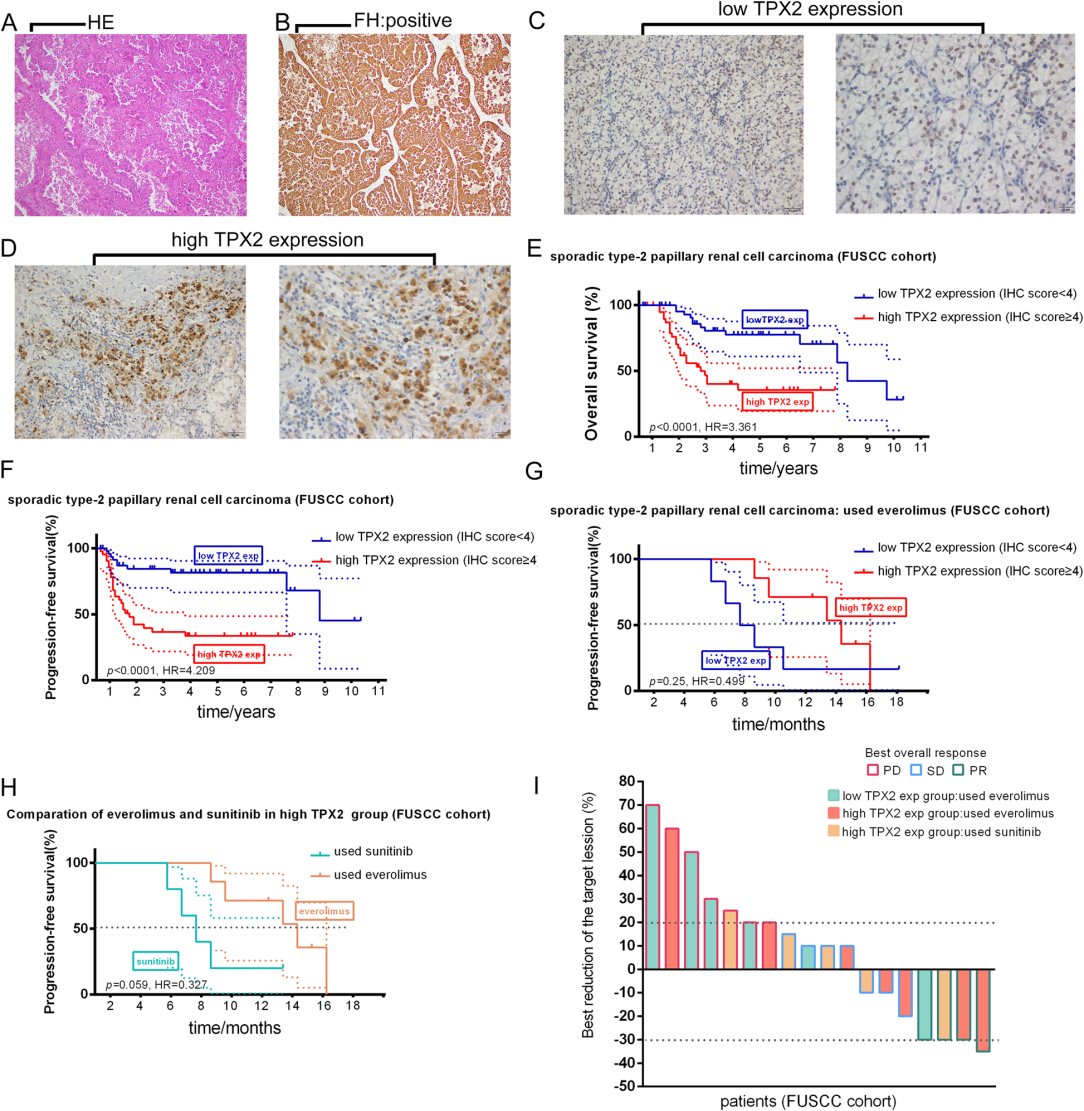
HE staining and FH staining of PRCC2 (**A, B**). Representative images of low and high TPX2 expression in sPRCC2 (**C, D**). Overall survival curve and progression-free survival of TPX2 expression in FUSCC cohort (**E–F**). Progression-free survival curve of sPRCC2 patients treated with Everolimus in FUSCC cohort (**G**). Progression-free survival curve of high risk sPRCC2 patients treated with Sunitinib or Everolimus (**H**). Tumor reduction image of sPRCC2 patients in FUSCC cohort

GSEA indicated (Fig. 7A-G) that higher TPX2 expression may be associated with G2M checkpoint, E2F targets, MYC targets, MTORC1 signaling, etc. Ninety-two patients with histopathologically confirmed sPRCC2 (positive staining for FH) were included (Fig. 8A-B), and representative images of TPX2 expression (IHC, low and high) are presented in Fig. 8C–D. Survival analysis indicated that both OS (HR = 3.361, *p* < 0.0001, Fig. 8E) and PFS (HR = 4.209, *p* < 0.0001, Fig. 8F) were significantly worse in the high-risk group than in the low-risk group. C-indices of prognostic models in FUSCC cohort indicated that TPX2 and KIF20A could serve as independent biomarkers (Table 5). Although not statistically significant, among patients who received first-line everolimus therapy, PFS was better in the high-risk group (*N* = 7) than in the low-risk group (*N* = 6, Fig. 8G). A retrospective analysis also indicated that everolimus exhibited better efficacy (Fig. 8H) in the high-risk group than sunitinib (*N* = 5). In summary, everolimus displayed greater efficacy in the high-risk group than in the low-risk group (overall response rate: 28.6% vs. 16.7%), and everolimus had greater efficacy than sunitinib in the high-risk group, including a better overall response rate (28.6% vs. 20%) and greater reduction of the target lesion (Fig. 8I).

**Table 5:** C-indices of prognostic models in FUSCC cohort

	overall survival		progression-free survival	
variables	c-index	se	c-index	se
T stage + N stage + M stage	0.75884839	0.03849097	0.73817941	0.0449859
T stage + N stage + M stage + TPX2 expression	0.78711041	0.03649335	0.77330977	0.0358449
T stage + N stage + M stage + KIF20A expression	0.7844691	0.03811392	0.75320371	0.0418312

*se* Standard error

## Discussion

Over these years, bioinformation has been applied for predicting kinds of biomarkers in cancers [35–37]. The present study focused on exploring the prognostic and therapeutic significance of TPX2 in PRCC2. Patients were stratified into high and low risk group according TPX2 expression. The high-risk group had a significantly worse prognosis than the low-risk group concerning both OS and DFS. The GSEA results indicated that compared with the findings in the low-risk group, gene expression in the high-risk group was significantly enriched in the mTORC1 signaling pathway, which may shed light on the use of mTOR inhibitors. In the external validation, TPX2 expression also revealed its strong ability to predict OS and PFS in the FUSCC cohort. The stronger efficacy effect of everolimus in the high-risk sPRCC2 group, although not statistically significant, exceeded our expectation.

TPX2 is a microtubule-associated protein that regulates the key step of spindle formation in mitosis, and modulates chromosomal structure [38]. TPX2 acts as different mechanisms in various tumors. Zhu D et al. [39] demonstrated that TPX2 inhibited tumor growth in osteosarcoma and high levels of TPX2 predicted poor prognosis for patients. They found that the mechanism involved is that TPX2 was negatively regulated by upstream miR-29c-3p, subsequently affected the downstream PI3K/AKT signaling pathway, thereby influenced the outcome. In some other cancers, such as cholangiocarcinoma [40] and prostate cancer [41], TPX2 is considered to be an essential factor for tumor development. However, Wang X et al. [42] elucidated the tight association between TPX2 and infiltrating CD8 + T cells in hepatocellular carcinoma(HCC). Contrary to other tumors, they used in vivo and in vitro experiments to demonstrate that TPX2 played an inhibitory role in HCC, which was accomplished by regulating the NF-κB signaling pathway and the expression of CXCR5 of infiltrating CD8 + T cells in HCC. Thus, they concluded that the TPX2 in HCC could be a potential target for anti-PD-1 therapy. Therefore, we can see that TPX2 can indicate the diagnosis, treatment and prognosis of a variety of tumors. The TME includes the function or metabolism of the host tissue, and the intrinsic environment of the cells, TME is regarded as combinations of immune cells, cancer-associated fibroblasts, endothelial cells, adjacent normal cells, etc. [43]. Overall, for non-clear cell renal cell carcinoma (nccRCC), a single-cell genomics study [44] demonstrated comprehensive expression and changes of immune cells, molecules, and related markers in the TME of nccRCC. It is also found that the heterogeneity of TME caused distinctly different biological features of nccRCC. Importantly, plenty of infiltrating cells and related genes formed different immunity status, therefore, focusing on these targets would have considerable implications for the clinical outcomes of pRCC [45]. In view of the property of high immune infiltration as mentioned before, immune-checkpoint inhibitor (ICI)-based therapy is considered as the effective treatment of metastatic renal cell carcinoma (mRCC) [46], for moderate and higher risk RCC, dual ICI/ICI could be regarded as the optimal choice of first-line treatments, ICI combined with VEGF or other therapy is available, too [47]. Based on the above viewpoints, while the risk classification by the expression of TPX2 has been accomplished, treatment including ICI combined with other classical therapies may be applied to the advanced RCC, that is, TPX2 could provide guidance for the clinical use.

We’ve also found another significant protein named KIF20A in high risk sPRCC2, it is also known as mitotic kinesin-like protein 2 (MKLP2), belonged to motor proteins, which plays an important role in promoting cytoplasmic division [48]. KIF20A is closely associated with a series of tumors, proteomic mapping displayed that KIF20A determined the aggregation of centrosomes in cancer cells, leading to apoptosis [49]. Besides, several bioinformatics studies have shown that KIF20A is a type of glycolytic gene and has an important predictive value for the diagnosis as well as the prognosis of tumors, such as hepatocellular carcinoma [50], retinoblastoma [51], cervical cancer [52], and breast cancer [53]. For renal cell carcinoma, KIF20A was regarded as promoting proliferation, invasion and migration of ccRCC [54], our study proved the carcinogenic property in sPRCC2 from our cohort.

Previous studies demonstrated that PRCC2 represents a heterogeneous group of lesions that can be divided into various subtypes according to genetic and molecular patterns, and these patterns reflect differences in the clinical course and prognosis of the disease. In a previous study [55], comprehensive genomic profiling was performed to sequence 315 genes, and the commonly altered genes in PRCC2 were CDKN2A/B (18%), TERT (18%), NF2 (13%), and FH (13%). Yang et al. [56] identified two highly distinct molecular PRCC subclasses via morphologic correlation, and they found that G1-S and G2-M checkpoint genes were dysregulated in class 1 and class 2 tumors. A similar pattern was observed in this research. We found that gene expression in high-risk sPRCC2 was enriched in the G2M checkpoint pathway (Fig. 7B), which suggests that the G2M checkpoint plays a key role in the malignant phenotype of sPRCC2. In 2016, the TCGA research network [8] revealed that PRCC2 can be further classified into three individual subgroups based on molecular differences associated with patient survival. Deng R et al. [57] have initially predicted molecular markers associated with immune infiltration in PRCC, furthermore, we explored biomarkers for type 2 of PRCC, which, after all, has a worse prognosis compared to type 1 of PRCC. In this research, we found that TPX2 expression could stratify sPRCC2 patients into high and low risk group and predict patients’ prognosis. Hua Z, et al. [58] found that M2 macrophages showed positive correlation with risk score, while M1 macrophages were the opposite. Their findings are inconsistent with ours, we checked the data and found the reason. As is known that type 2 pRCC has a distinct worse outcome than type 1 pRCC [9, 10]. Hua’s research contains both type 1 and type 2 pRCC, while our research only focused on type 2 pRCC. And type 2 pRCC has a more heterogenous spectrum of chromosomal gains and losses, thus the M1 macrphages may take a different role in type 2 pRCC.

Our results demonstrated that high-risk sPRCC2 was significantly correlated with a higher tumor stage, a higher lymph node stage, and worse OS. Because the treatment of advanced PRCC2 remains difficult, it is of great importance to identify potential targets suppressing PRCC2 growth. As mentioned in a previous study [8], the classification of PRCC may have a significant impact on clinical and therapeutic management and clinical trial design. Mutation of NF2 (the Hippo pathway tumor suppressor) was observed in a number of PRCCs, and this pathway has been targeted in other cancers [59]. The NRF2–ARE pathway was upregulated in both hereditary PRCC and sPRCC2. Currently, researchers are interested in the NRF2–ARE pathway, and novel strategies targeting this pathway have recently been developed [60, 61]. In this study, we found that high-risk sPRCC2 exhibited excellent mTORC1 signaling pathway activity, which suggests the potential accurate use of mTOR inhibitors. Everolimus, an oral mammalian mTOR inhibitor, has antitumor activity in multiple cancer types [62], and previous research demonstrated that everolimus has some clinical benefit in patients with metastatic PRCC [63, 64]. In our retrospective analysis of the FUSCC cohort, everolimus exhibited a stronger drug effect against high-risk sPRCC2 than against low-risk sPRCC2, and everolimus had greater activity in the high-risk group than sunitinib. This result conferred that the TPX2 expression can also guide the accurate use of everolimus in sPRCC2.

This study had several limitations. The nature of retrospective research limits the clinical value of this work. Further validation in multicenter or prospective studies is needed to verify the findings. However, it is difficult to conduct randomized controlled trials in sPRCC2 because of its rarity. There is also an urgent need for in vitro and in vivo experiments to explore the underlying mechanisms.

## Conclusion

TPX2 was a prognostic and therapeutic biomarker in PRCC2. Higher abundance of tumor infiltrating M1 macrophage was significantly associated with worse overall survival in PRCC2. mTOR inhibitors may have good efficacy in patients with high-risk PRCC2.

## Supplementary Information

**Additional file 1:** **Table S1.** Down-regulated and up-regulated differentially expressed genes in three cohorts.

**Additional file 2: **Table S2. Detail clinical information of 92 sPRCC2 from FUSCC cohort.

**Additional file 3: Table S3.** Estimation of drug effect in FUSCC cohort.

**Additional file 4 **.

## Data Availability

GSE26574 data sets was obtained from https://www.ncbi.nlm.nih.gov/geo/query/acc.cgi?acc=GSE26574. GSE48352 data sets was obtained from https://www.ncbi.nlm.nih.gov/geo/query/acc.cgi?acc=GSE48352. Gene expression profiles and clinical information of PRCC from TCGA were downloaded from https://portal.gdc.cancer.gov/. The data from FUSCC cohort during the current study available from the corresponding author on reasonable request.

## Abbreviations

RCC: Renal cell carcinoma
ccRCC: Clear cell renal cell carcinoma
nccRCC: Non–clear cell renal cell carcinoma
PRCC: Papillary renal cell carcinoma
PRCC1: Type 1 papillary renal cell carcinoma
PRCC2: Type 2 papillary renal cell carcinoma
TCGA: From the Cancer Genome Atlas
FUSCC: Fudan University Shanghai Cancer Center
GEO: Gene Expression Omnibus
GSEA: Gene set enrichment analysis
